# Genome-wide 5-hydroxymethylcytosine (5hmC) reassigned in Pten-depleted mESCs along neural differentiation

**DOI:** 10.3389/fcell.2022.956604

**Published:** 2022-12-22

**Authors:** Zhangting Wang, Kai-Kei Miu, See-Wing Chan, Fanghong Ou, Patrick Wai-Nok Law, Wai-Yee Chan

**Affiliations:** ^1^ School of Biomedical Sciences, Faculty of Medicine, The Chinese University of Hong Kong, Hong Kong SAR, China; ^2^ Key Laboratory for Regenerative Medicine, Jinan University-The Chinese University of Hong Kong, Ministry of Education, School of Biomedical Sciences, Faculty of Medicine, The Chinese University of Hong Kong, Hong Kong SAR, China

**Keywords:** midbrain/hindbrain progenitors, Pten, 5-hydroxymethylcytosine, nano-5hmC-seal, mitochondrial respiratory chain

## Abstract

DNA methylation and hydroxymethylation have been implicated in the regulatory dynamics of gene expression in normal development and differentiation. 5-Hydroxymethylcytosine (5hmC), created by the ten-eleven translocation (TET) protein-catalyzed oxidation of 5-methylcytosine (5mC), is abundant in the brain, but the genome-wide distribution and impact of 5hmC during diverse neuronal differentiation remain unknown. Here, we used an *in vitro* model to differentiate mouse embryonic stem cells (mESCs) into ventral midbrain and hindbrain neural progenitors, followed by characterizing global 5hmC distribution using a nano-5hmC-seal approach. The 5hmC pattern was dynamic in promoter, exon, and enhancer regions, associated with gene activation and repression. For example, ventral midbrain markers (*Lmx1a*, *Otx2*, and *Th*) and hindbrain markers (*Hoxa1*, *Zic1*, and *Tph1*) acquire 5hmC and are upregulated during differentiation. Among the differentially expressed genes involved in both midbrain and hindbrain lineage commitment, phosphatase and tensin homolog (Pten) was identified as a key regulator for neuronal development. We confirmed that Pten knockout disrupted the normal differentiation of midbrain/hindbrain neural progenitors, resulting in immature neurons. In addition, 5421 and 4624 differentially hydroxymethylated regions (DhMRs) were identified in the differentiation of Pten^−/−^ mESC into ventral midbrain and hindbrain progenitors, respectively. Gene ontology analysis showed that the majority of these DhMRs were associated with neurogenesis, ectoderm development, and signal transduction. Moreover, further combinational analysis of the 5hmC pattern and transcriptomic profile in the midbrain progenitor cells demonstrated Pten as a toggle to modulate mitochondrial associated pathways. Therefore, our findings elucidated the molecular mechanisms underlying lineage-specific differentiation of pluripotent stem cells to the midbrain/hindbrain progenitors, where Pten participates as one key regulator.

## 1 Introduction

Neural development and differentiation, despite being complex in nature, are highly regulated processes coordinated by location-specific morphogenic cues, each defining stages and the spatiotemporal changes along the development. These cues were later discovered to instruct dynamic changes in heterochromatin reorganization, conforming to DNA and histone protein modifications that open up a concordant gene profile to establish cell identity in normal biological processes and diseases (Szulwach et al., 2011; Kazakevych et al., 2017; Rodríguez-Aguilera et al., 2020). The most common modification in DNA is active methylation of cytosine at clustered CpG sequences to control developmental processes. The dynamic methylation and demethylation of cytosine paved another level of plasticity in gene expression, resulting in epigenetic changes. Such removal of cytosine methylation requires an intermediary but relatively stable hydroxyl modification in the methyl group, that is, 5-hydroxymethylcytosine (5hmC) state before 5-methylcytosine (5mC) is removed, and such a DNA demethylation process is named as a gene activation mechanism at certain developmental stages (Breiling and Lyko, 2015).

The ten-eleven translation (TET)-family of oxidative enzymes catalyzes 5mC to 5hmC resulting in this marker as the *de facto* recognition cue by the base excision DNA repair machinery, and this process is coined as ‘active cytosine demethylation’ (Ito et al., 2010). This intermediate step along unmodified cytosine restoration is therefore presumed as the predominant mechanism over passive demethylation with definable assumes functional roles, especially favored for the central nervous system (CNS) development (Spiers et al., 2017; Antunes et al., 2019). Nevertheless, the transient base change received new attention along the discovery of its specific molecular readers of 5hmC, such as UHRF2, layering post-translational control to furnish a unified molecular pathway comprised of transcriptional regulators in coerced actions with some other DNA damage and repair proteins (Mellén et al., 2012; Chen et al., 2017).

It was suggested that pro-active dynamic reassignment of 5hmC likely evoked an acquired “imprint” of cell identity favoring adult progenitor cell differentiation in major organs such as the liver and brain (Rodríguez-Aguilera et al., 2020; Kuehner et al., 2021; MacArthur and Dawlaty, 2021). Intriguingly, despite being a transient mark, the global 5hmC level persists in the mature CNS, and it was determined to cluster around the vicinity of gene loci bearing synapse functions. Similarly, the same mark was found to span across cis-regulatory elements for key developmental genes within both murine and human fetal brains, followed by surging levels postnatally along this key stage of neuronal maturation with profound synaptogenesis (Spiers et al., 2017; He et al., 2021). Notably, novel evidence pinpointed dysregulation of 5hmC levels that was implicated in the etiology of psychiatric and neurodevelopmental disorders, not limited to autism spectrum disorders and schizophrenia (Cheng et al., 2018). Unfortunately, despite the fact that a surveillance profile for 5hmC distribution along adult progenitor cell fate commitment had already been performed in several human tissues, the frequent lack of base resolution limited by those obsolete profiling methods hindered effective prediction of the actions of these dynamic marker changes.

Phosphatase and tensin homolog (PTEN) was first described as a tumor suppressor gene in gliomas (Zheng et al., 2008). The same gene is implicated in genetic susceptibility to related diseases, where PTEN germline mutations are associated with Bannayan–Zonana syndrome, Cowden disease, and Lhermitte–Duclos disease, all manifested as disorganized hamartomas in multiple organs (Liaw et al., 1997; Marsh et al., 1998). Some of the patients showed inborn disorders in neural development, including macrocephaly and cerebellar hypertrophy, which manifested as intellectual disability and seizures. Glial and neuronal-specific deletion of PTEN at embryonic day 9.5 (E9.5) within the midbrain–hindbrain junction area where the natural isthmus organizer resides had proven to result in ataxia and reduced neuronal activity (Backman et al., 2001).

In this study, we sought to characterize the dynamic 5hmC landscape of both differentiating midbrain and hindbrain progenitors and establish its correlation with gene expression changes. Moreover, we confirmed that Pten, a pleotropic developmental gene, is being highly expressed under these contexts to participate in the differentiation of mouse embryonic stem cells (mESCs) towards their commitment into either midbrain or hindbrain identity, likely guiding the dynamic 5hmC changes concurrently. Interestingly, loss of Pten resulted in diminished differentiation potential to commit towards mature cell fates as a result of disrupted mitochondrial functions.

## 2 Materials and methods

### 2.1 Mouse ESC culture

The mouse E14 cell line was obtained from the American Type Culture Collection (ATCC, ES-D3, CRL-1934). The mESC Pten^−/−^ cell lines were previously generated in our lab using CRISPR-Cas9 (Wang et al., 2020). The cells were cultured in DMEM/F12 medium supplemented with 15% fetal bovine serum (Hyclone, SH30071), GlutaMAX, non-essential amino acid solution, sodium pyruvate (Life Technologies, 10565), and β-mercaptoethanol (Life Technologies, 31350). Small molecules or morphogen, including LIF (Millipore, ESG1106), CHIR99021 (Sigma, SML1046), and PD0325901 (Sigma, PZ0162) were added into the medium. A mycoplasma contamination test was performed for these cell lines.

### 2.2 Neuronal differentiation of mESCs

**FIGURE 1:**
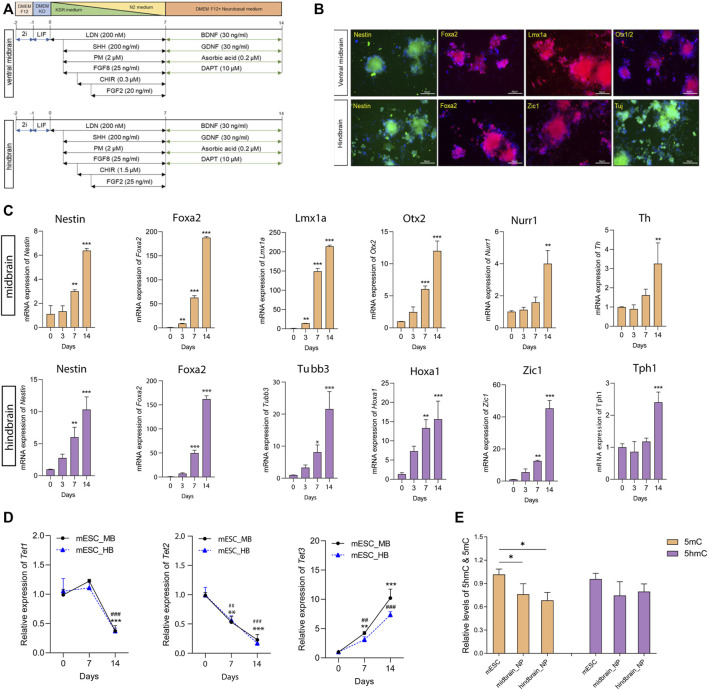
Differentiation of mouse ESCs into ventral midbrain and hindbrain fate. **(A)** A schematic overview of the protocol for the differentiation of mouse ESC into ventral midbrain (MB) and hindbrain (HB) progenitors. **(B)** Representative photos depicting expression of MB markers (Nestin, Foxa2, Lmx1a, and Otx1/2) and HB markers (Nestin, Foxa2, Zic1, and Tuj). **(C)** Transcriptional expression of specific neuronal marks in MB and HB specifications. **(D)** qRT-PCR of *Tets* in the three differentiation states. **(E)** Relative global 5mC and 5hmC levels in different differentiation states, measured by LC-MS/MS. All data are expressed as mean ± SD (*n* = 3). *p < 0.05, **p < 0.01, and ***p < 0.001; ^#^ *p* < 0.05, ^##^ *p* < 0.01, and ^###^ *p* < 0.001.

Monolayer culture for neuronal differentiation was performed to generate neural progenitors based on a well-characterized protocol with minor modifications (Alsanie et al., 2017). In brief, cells were dissociated and plated at a density of 1 × 10^4^ cells/cm^2^ in culture medium. For the ventral midbrain specification, patterning medium containing SHH (200 ng/ml), FGF8 (25 ng/ml), PM (2 µM), and LDN (100 nM) were used from day 1 to day 7. The patterning medium was further supplied with CHIR9902 (0.3 µM) and FGF2 (20 ng/ml) from day 2 and day 3, respectively. From days 7–14, medium was replaced with maturation medium. Similarly, for the hindbrain lineage, patterning medium supplemented with SHH (200 ng/ml), FGF8 (25 ng/ml), PM (2 µM), and LDN (100 nM) was applied from day 1 to day 7. CHIR9902 (1.5 µM) was added to the medium at day 2, and additional FGF2 (20 ng/ml) was added to the patterning medium at day 3. All patterning medium was changed every day for the first week, and maturation medium was changed every 2 days until day 14 (Figure 1A).

### 2.3 RNA isolation and real-time PCR

To extract total RNA from cells, a standard protocol with TRIzol reagent (Invitrogen, United States) was applied. RNA concentration and quality were assessed by the NanoDrop 2000 (Thermo Fisher Scientific, United States). The reverse transcription PCR and real-time PCR were performed with the PrimeScript^TM^ Reverse Transcriptase kit (Takara, Japan) and ABI ViiA 7 Real-Time PCR System (Applied Biosystems, Life Technologies, United States). SYBR Green real-time PCR kit (Applied Biosystems, Life Technologies, United States) was used for real-time PCR. Relative expression of genes was analyzed by 2^-△△Ct^ method, with *GAPDH* as the housekeeping gene. The sequence of each primer is shown in Supplementary Table S1.

### 2.4 Immunostaining

The immunostaining was performed according to a previous protocol (Wang et al., 2022). Briefly, cells were fixed in 4% formaldehyde and permeabilized with 0.2% Triton X-100. The cells were then blocked with 2% goat serum and incubated with primary antibodies overnight at 4°C. After washing three times with PBS, cells were then incubated with secondary antibodies for 1 h at room temperature. Hoechst 33342 (Life Technologies, United States) was used to stain the nucleus. Images were obtained using the Nikon Live Cell Imaging System Ti-E inverted fluorescence microscope.

### 2.5 5hmC and 5mC quantification by LC-MS/MS

The 5hmC and 5mC content in cells were quantified by the LC-MS/MS using a stable isotope-labeled internal standard as described previously (Le et al., 2011; Zhang et al., 2016). Briefly, genomic DNA was extracted using DNA extraction kits (Life Technologies, United States) according to the manufacturer’s protocol. Enzymatic digestion of DNA was performed with dsDNA Shearase (Zymo Research, United States). The nucleoside mixture from DNA was oxidized in a buffer containing acetonitrile, formic acid, and MnO_2_ and incubated at 40°C for 1 h. The mixture was then purified by a carbon black-SPE column to remove salt, followed by derivatization with dansylhydrazine (DNSH). The labeled products were then reconstituted in water and subjected to UHPLC-MS/MS analysis.

The UHPLC-MS/MS analysis was performed on an Agilent 6460 Triple Quadrupole LC/MS System with a Waters Acquity BEH C18 column. A mobile phase consistent of A (water containing 0.1% formic acid) and B (acetonitrile containing 0.1% formic acid) was used following a gradient elution protocol. The follow rate was set as 0.3 ml/min with 3-µl injection volume. The multiple reaction monitor was used for the mass spectrometer with m/z 242.1→126.1 for 5mC, 258.1→142.1 for 5hmC, and 228.1→112.1 for dC. Collision energy and fragmentor were 33 V and 185 V, respectively.

### 2.6 Nano-5hmC-seal

The library preparation of 5hmC was performed according to a previously described method with modifications (Han et al., 2016). The genomic DNA was extracted using PureLink® Genomic DNA Kits (Invitrogen, United States). Tagmentation and purification steps were performed using the TruePrep DNA Library Prep Kit V2 for Illumina (TD501, Vazyme, China) and AMPure XP beads (Beckman Coulter, United States). The glucosylation reaction was performed in a mixture buffer containing 50 mM HEPES buffer (pH 8.0), 25 mM MgCl_2_, purified DNA, 100 µM N3-UDP-Glc, and 1 µM βGT. After 1 h of incubation, DBCO-PEG4-DBCO was added to the reaction mixture for further incubation at RT. Finally, the modified DNA was purified and incubated with C1 streptavidin beads (Life Technologies, United States) at RT for 30 min. After being washed five times, the captured DNA fragments were eluted and underwent PCR amplification using the TruePrep DNA Library Prep Kit. The concentration of each DNA library was measured with a Qubit fluorometer (Life Technologies, United States). Sequencing was performed on the HiSeq platform from Illumina.

### 2.7 Bioinformatical analysis

All the processed raw reads were aligned to the mouse mm10 assembly using the Bowtie program and SAMtools to generate the bam and bedGraph files (Li et al., 2009). Distribution of 5hmC regions/peaks was characterized with MACS2 (Zhang et al., 2008). Comparing among samples was performed by the MAnorm (Shao et al., 2012) using files generated from MACS2. Heatmap of peak distribution and K-means clustering were obtained using deepTools software. The Bioconductor DESeq2 packages were used for the comparison between each sample (Love et al., 2014). Functional annotation of DhMRs was obtained with Metascape (Zhou et al., 2019). GO term analyses were performed by DAVID and visualized as a functional gene network (Sherman et al., 2022).

### 2.8 Statistical analysis

All data were presented as mean ± standard. Statistical analysis was performed by GraphPad Prism 9 (GraphPad Software Inc., San Diego, CA). The comparison between two groups was assessed by a Student’s *t*-test, and a one-way ANOVA followed by a *post hoc* test was used for the comparison among multiple groups. *p* < 0.05, *p* < 0.01, or *p* < 0.001 was identified as statistically significant.

## 3 Results

### 3.1 Differentiation of mESC into midbrain/hindbrain neurons

During embryonic development, the transient isthmic organizer situated in the midbrain–hindbrain boundary actively secretes FGF8 and Wnt ligands to modulate the development of ventral midbrain (VM) progenitors and neurons (Brodski et al., 2019). To study the role played by DNA hydroxymethylation along specified lineage commitments, mESC cells were subjected to lineage-specific differentiation to form ventral midbrain (MB) and hindbrain (HB) neurons *in vitro* according to well-modified protocols (Figure 1A) (Alsanie et al., 2017). High levels of the regional identity proteins FoxA2 and the forebrain–midbrain marker Otx1/2 were detected in the ventral midbrain progenitors (Figure 1B). Furthermore, the essential MB dopaminergic transcripts (*Lmx1a*, *Nurr1*, *and Th*) were upregulated during differentiation toward MB progenitors (Figure 1C). Moreover, in mESC-derived hindbrain progenitors, a 7-day culture model elegantly demonstrated profound induction in the transcript levels of two critical hindbrain patterning genes: *Hoxa1*, a critical effector gene for the early patterning of the rhombencephalon, and *Zic1*, a key regulator responsible for the proliferation of progenitors during early cerebellar development (Figure 1C). In the hindbrain lineage maturation, 5-hydroxytryptamine positive serotonergic neurons were enriched with high expression of *Tph1* (Figure 1C).

Next, we determined the expression levels of Tets and DNA methyltransferases (Dnmts) along differentiation. Among the Tet enzymes, Tet1 was found to dissipate along maturation; *Tet2* expression decreased while *Tet3* increased during the differentiation (Figure 1D). In the two Dnmts, both *Dnmt3a* and *Dnmt3b* increased during patterning and maturation stages in cells committed hindbrain lineage. However, in the midbrain lineage, the two Dnmts were highly expressed in the neural progenitor cells, but their expression was reduced along maturation (Supplementary Figure S1A). The alteration of Tets and Dnmts paved our to examine the global change of DNA methylation and hydroxymethylation along its differentiation. Therefore, we performed a UHPLC-MS/MS-based method to ascertain the 5mC and 5hmC content in mESC-derived midbrain and hindbrain neurons (Supplementary Figure S1B). Results showed that the global 5mC level significantly decreased during differentiation when compared to the pluripotent stages (Figure 1E).

### 3.2 Differentially hydroxymethylated regions associated with neural differentiation

**FIGURE 2:**
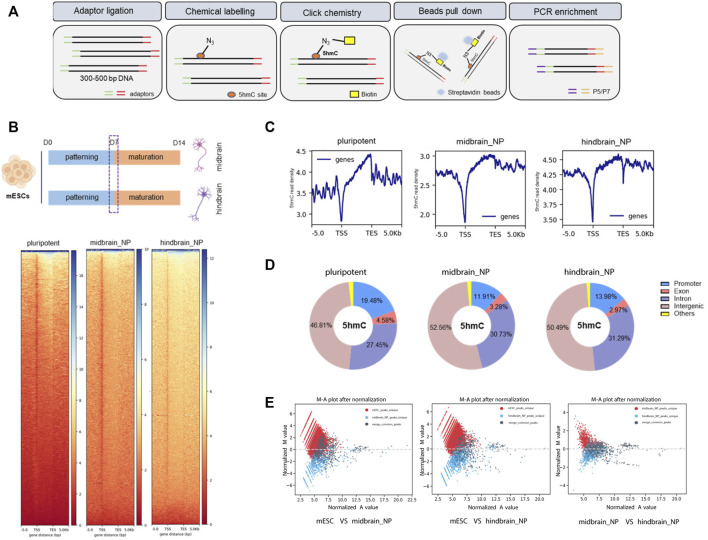
Characterization of global 5hmC distribution in midbrain and hindbrain progenitor cells. **(A)** Schematic steps for nano-5hmC-seal approach. **(B)** Heatmap representation of 5hmC densities within 5 kb of transcription start site (TSS) and transcription end site (TES) in midbrain and hindbrain progenitor cells as a function of gene expression of level. **(C)** Metagene profile for genes in pluripotent mESCs, midbrain, and hindbrain progenitors, from 5 kb upstream of the TSS to 5 kb downstream of the TES. **(D)** Genomic distribution of 5hmC-enriched regions in progenitor cells. Promoters were defined as ± 5 kb from the annotated TSS. **(E)** MA plot of 5hmC peaks in midbrain/hindbrain progenitor cells and mouse ESCs after normalized by total reads using MAnorm normalization models.

The nano-5hmC-seal method is a highly selective chemical labeling protocol to capture genome-wide 5hmC (Figure 2A) (Han et al., 2016). From all our adopted *in vitro* protocols, it was defined that the transcriptome shall readily reflect the scheduled activities of neural progenitor within a week of directed neural differentiation; therefore, we picked this time point to assess 5hmC occupancy and dynamics across different differentiation lineage, namely, midbrain and hindbrain progenitor cells. We identified 22,551 and 21,147 differentially hydroxymethylated regions (DhMRs) during differentiation of mESC cells into midbrain and hindbrain progenitors, respectively. The 5hmC distribution was distinct during neural progenitor differentiation (Figure 2B), with most of the identified peaks enriched at promoter, intron, and intergenic regions (Figures 2C,D). The distribution of transcription factor-binding loci relative to the transcription start site (TSS) in each group was also profiled (Supplementary Figure S2A). During neural differentiation, activated genes acquired 5hmC near the transcription start site (TSS), while repressed genes showed a loss of 5hmC. For example, the pluripotency-related gene Nanog showed loss of 5hmC in the closet enhancer and gene body, whereas neural differentiation markers acquired 5hmC in near the TSS (Supplementary Figure S2B), indicating a dynamic change of 5hmC during neurogenesis. Compared to the pluripotent state, more than half of the 5hmC changes occurred in gene bodies and promoters (Supplementary Figure S2C). Additionally, among the identified 5hmC regions, 796 and 1,047 peaks were identified to be unique in the midbrain and hindbrain progenitors, respectively. However, when we further comparing the hindbrain and midbrain progenitors, 106 midbrain unique peaks and 398 hindbrain unique peaks were identified, indicating the different dynamic 5hmC changes along different differentiation lineage (Figure 2E).

**FIGURE 3:**
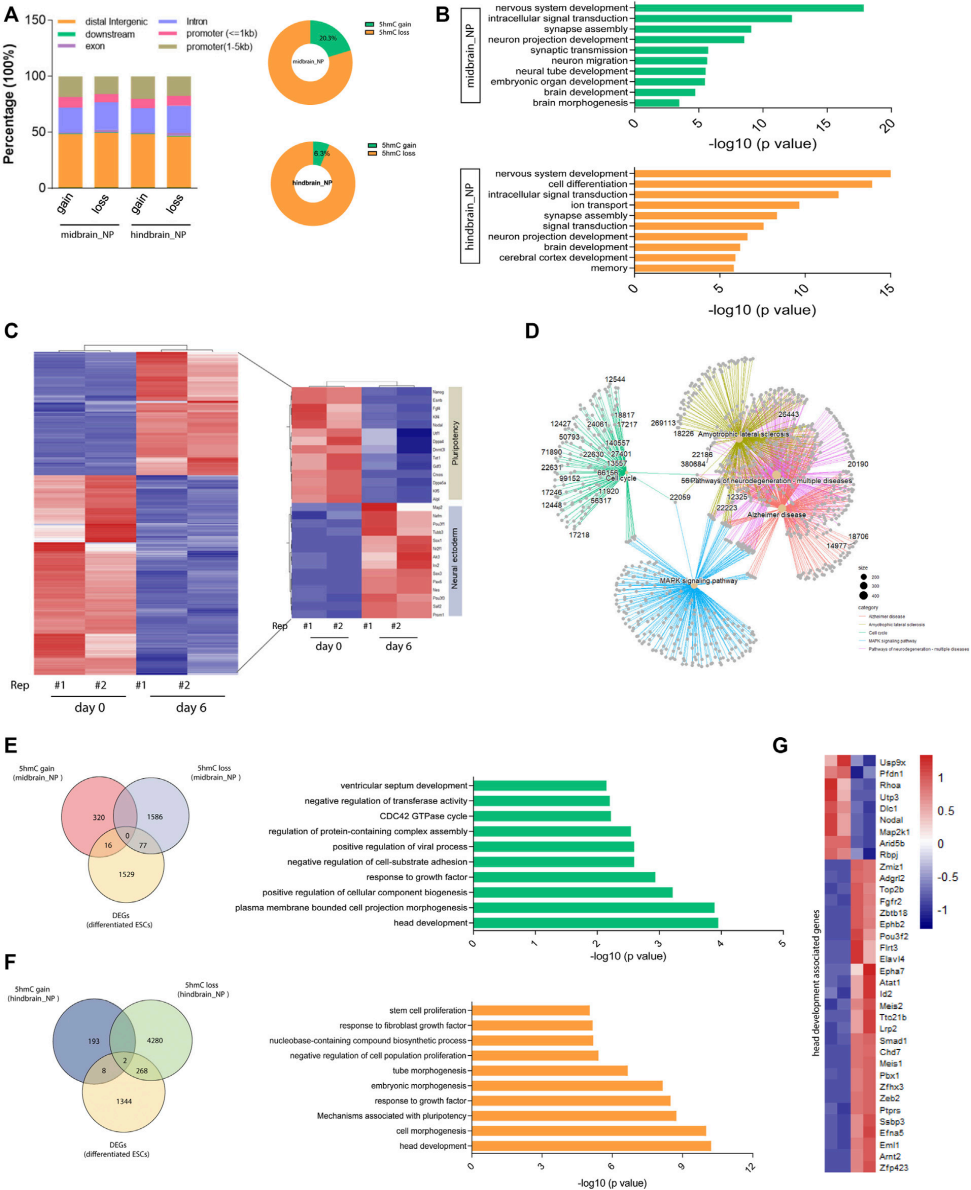
Differentially hydroxymethylated regions (DhMRs) associated with neural differentiation. **(A)** Genomic distribution of identified DhMRs in the differentiation of midbrain and hindbrain specification. **(B)** Gene ontology analysis for the DhMRs in the two progenitors, upper for the midbrain progenitors and lower for the hindbrain progenitors. **(C)** Hierarchical clustering of differentially expressed genes in mouse ESC at day 6 after neural differentiation. **(D)** Network plot of enriched terms colored by cluster identity. **(E)** DhMRs identified in the midbrain progenitors were overlapped with DEGs in the Venn diagram (left). Metascape analysis of these clustered DhMRs (16 5hmC gain and 77 5hmC loss) (right). **(F)** DhMRs identified in the hindbrain progenitors were overlapped with DEGs in the Venn diagram (left). Metascape analysis of these clustered DhMRs (eight 5hmC gain and 268 5hmC loss) (right). **(G)** Hierarchical clustering of genes associated with head development.

Further analysis found that most of the differentially hydroxymethylated regions (DhMRs) were located at their distal intergenic and promoter regions during differentiation (Figure 3A). Annotation of the DhMRs revealed that about 20% and 6.3% of the peaks showed 5hmC gain in midbrain and hindbrain differentiation lineages, respectively (Figure 3A). The Kyoto Encyclopedia of Genes and Genomes (KEGG) pathway analysis further demonstrated that most of the DhMRs in the midbrain_NP were associated with neural development and signal transduction (Figure 3B, upper). Similarly, DhMRs detected in the hindbrain progenitors were shown to be involved in the nervous system development, including synapse assembly, neuron projection, and cerebral cortex development (Figure 3B, lower).

To establish the actual linkage between 5hmC local enrichment and related gene expression, we compared the identity of the most proximal gene in the DhMRs with the list of differentially expressed genes (DEGs) during neurogenesis. We examined the transcriptomic profile of wild-type mESC at day 6 after neural differentiation (GEO: GSE: 95127) (Yao et al., 2018). A set of neural ectoderm genes were significantly upregulated during neural differentiation, with suppression of pluripotency markers (Figure 3C). KEGG analysis of these differentially expressed genes (DEGs) in the neural progenitor cells enriched pathways associated with neurodegenerative diseases, Alzheimer disease, and the MAPK signaling pathways (Figure 3D and Supplementary Figure S3A). We then further clustered these DEGs with DhMRs identified in the midbrain and hindbrain progenitors, respectively. It was shown that 93 DhMRs (including 16 5hmC gain and 77 5hmC loss peaks) identified in the midbrain progenitors were co-clustered with DEGs, and these genes were associated with head development and cellular signal transduction (Figure 3E and Supplementary Figure S3B). Similarly, a total of 278 DhMRs in the hindbrain progenitors overlapped with the DEGs of the neural progenitor cells, which are tightly linked to head development, embryonic morphogenesis, and cellular component biogenesis (Figure 3F and Supplementary Figure S3C). We then further analyzed the 36 genes associated with head development (Figure 3G) and found that most of them were reported to regulate the development of the hindbrain, forebrain, cerebellum, and limbic system (Supplementary Figure S3D).

### 3.3 Loss of Pten impaired mESC-derived neural differentiation

**FIGURE 4:**
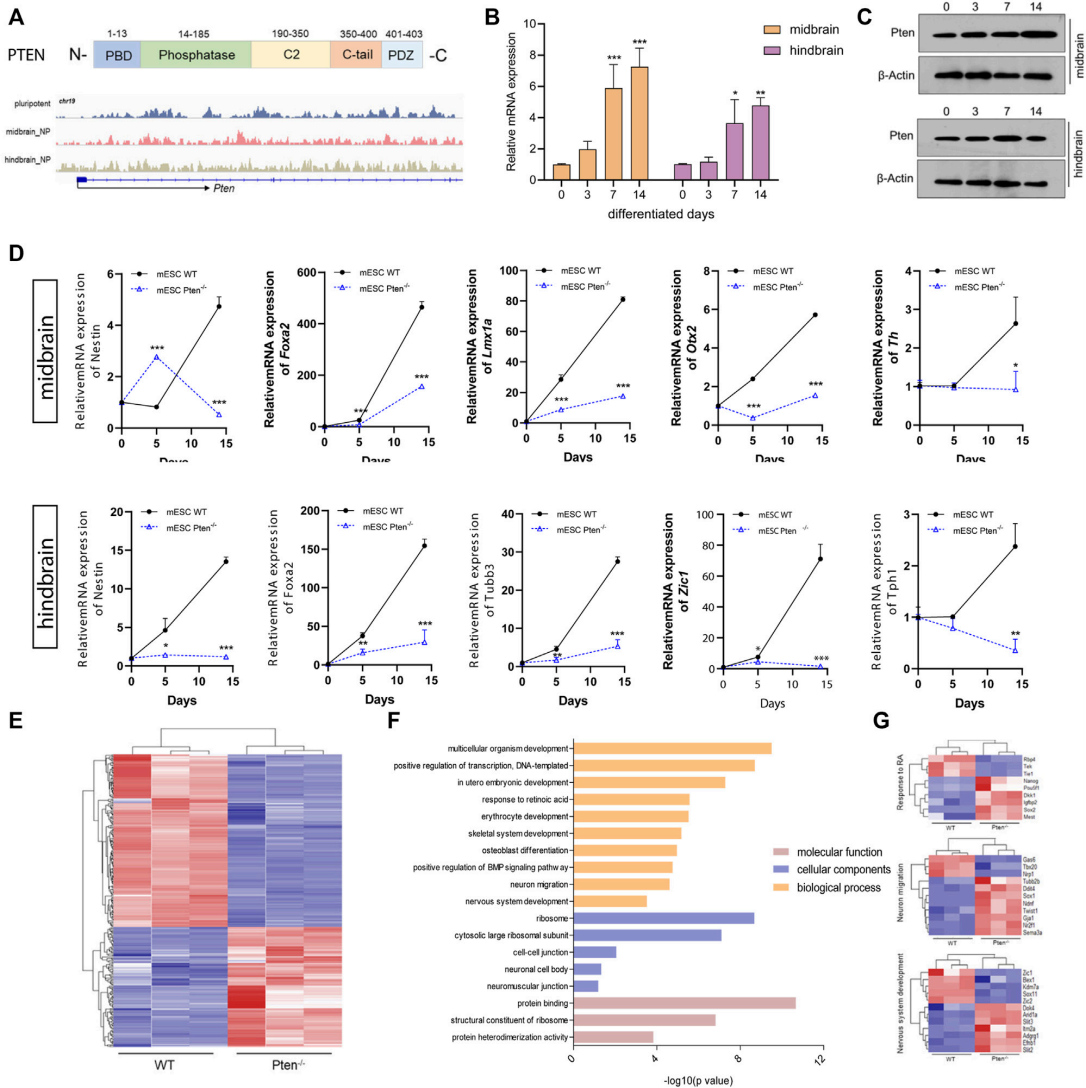
Loss of Pten disrupted neurogenesis towards midbrain and hindbrain specification. **(A)** Genome browser visualization of 5hmC patterns of Pten in the midbrain and hindbrain progenitors derived from wild type (WT) and Pten^−/−^ (KO) mESCs. The mRNA **(B)** and protein expression **(C)** of Pten in the differentiation of midbrain and hindbrain specification. **(D)** Transcriptional expression of midbrain and hindbrain markers in WT and KO ESCs. **(E)** Heatmap of pluripotency genes in WT and Pten^−/−^ ESCs. **(F)** Gene ontology analysis for the DEGs identified pathways associated with the nervous system development. **(G)** Selected DEGs responsible for the neuronal development. All data are expressed as mean ± SD (*n* = 3). *p < 0.05, **p < 0.01, and ***p < 0.001; ^#^ *p* < 0.05, ^##^ *p* < 0.01, and ^###^ *p* < 0.001.

The Pten gene is reputable for being a tumor suppressor gene, while it is also heavily involved in instructing either renewal or differentiation of neural and glioma stem/progenitor cells (Zheng et al., 2008). In our study, we examined that Pten gained 5hmC in both the enhancer and gene body in both mESC-derived MB and HB progenitor cells, with induction of both mRNA and protein expression levels along neural differentiation (Figures 4A–C). Since our lab has previously established a Pten^−/−^ mESC lines with the deletion of codon region using the CRIPSR-Cas9 system (Wang et al., 2020) (Supplementary Figure S4A), we therefore use this cell lines to investigate the role of Pten in regulating neurogenesis. Immunostaining confirmed that Pten was not expressed in the Pten^−/−^ mESCs (Supplementary Figure S4B). These cell lines were then differentiated toward midbrain and hindbrain neural progenitors according to the protocols shown in Figure 1A. As expected, the expression of the plethora of midbrain and hindbrain markers dwindled in Pten^−/−^ (KO) cells than wild-type (WT) cells (Figure 4D), suggesting that Pten deletion impaired the differentiation in neural lineage commitment.

We then compared gene expression profile datasets (GEO: GSE117280) (Wang et al., 2020) derived from EBs containing both WT and Pten^−/−^ ESC cells (Figure 4E). In the mESC-derived EBs, RNA-seq results identified a collection of DEGs, with 876 genes upregulated while 874 genes downregulated under Pten loss of function. The gene ontology (GO) analysis indicated a majority of these DEGs were involved in relevant developmental processes of neurogenesis, ectoderm development, and signal transduction (Figure 4F). On the other hand, there is also a strong hit in marker genes governing stem cell maintenance and differentiation (Figure 4F). A set of stem cell pluripotent markers including *Nanog*, *Pou5f1*, *Sox2*, *Sema3a*, and *Dkk1* was found to be disrupted by the loss of Pten during the differentiation of ESCs into EBs. In addition, decreased expression was observed for neural markers *Zic1* and *Zic2* in the Pten^−/−^ cell-derived EBs, reinforcing the critical role of Pten in the neurogenesis (Figure 4G).

### 3.4 Pten^−/−^-associated global DNA hydroxymethylation changes during neurogenesis

**FIGURE 5:**
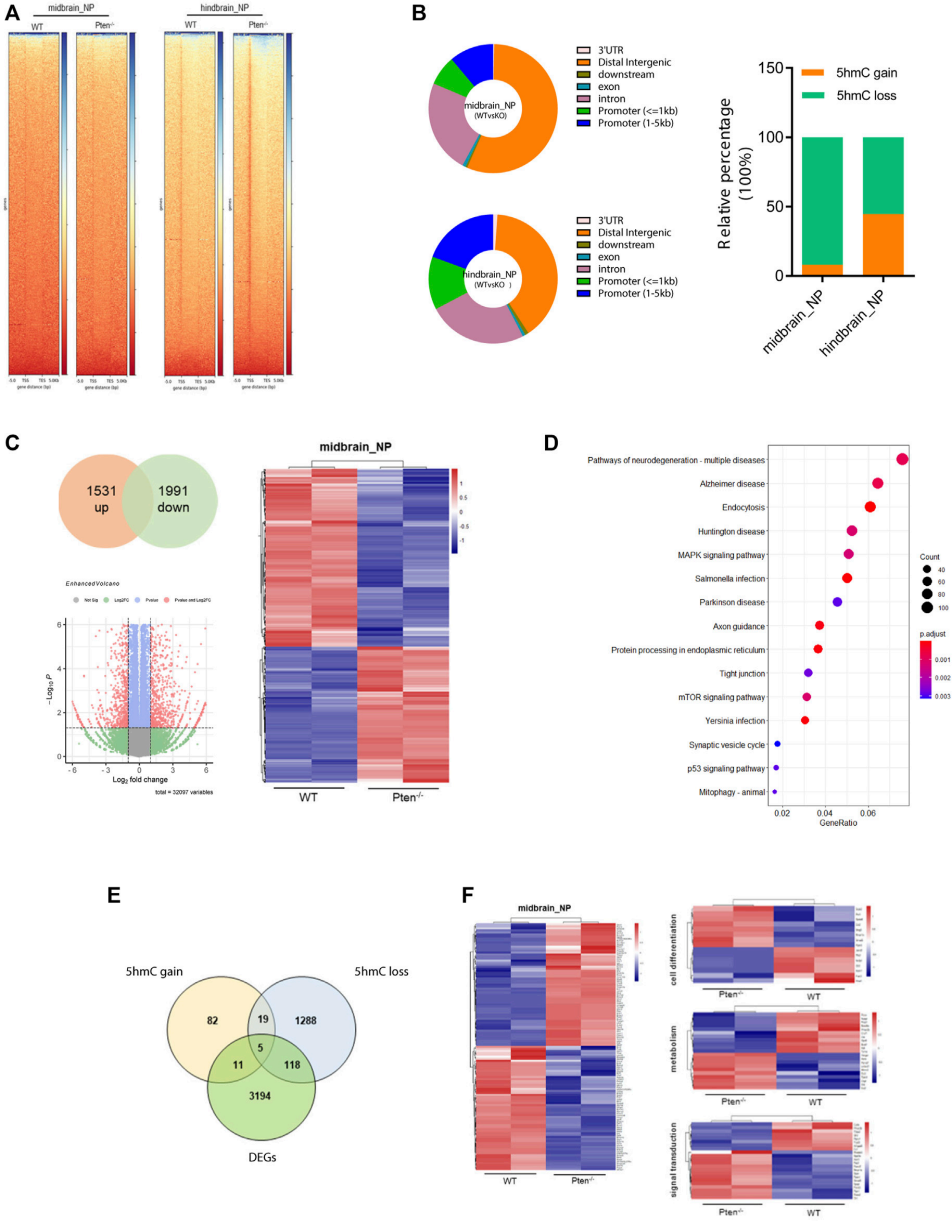
Pten^−/−^ associated global DNA hydroxymethylation changes during neurogenesis. **(A)** Heatmap representation of 5hmC densities within 5 kb of transcription start site (TSS) and transcription end site (TES) in WT and Pten^−/−^ ESCs in midbrain and hindbrain progenitor cells. **(B)** Genomic distribution of 5hmC-enriched regions in progenitor cells. **(C)** Transcriptional analysis of genes in WT and Pten^−/−^ ESCs in midbrain progenitor cells. Volcano plots (left) and heatmaps (right) were shown. **(D)** KEGG pathway enrichment analysis identified pathways associated with neurodegenerative diseases. **(E)** Venn diagram showing overlap of upregulated DhMRs, downregulated DhMRs and DEGs in WT and Pten^−/−^ ESCs-derived midbrain progenitor cells. **(F)** Heatmap of the 123 DEGs with 5hmC loss enriched pathways associated with cell differentiation, signal transduction, and regulation.

To test the functional role of Pten in DNA demethylation, we collected genomic DNA at differentiated day 7 from mESC-derived ventral midbrain and hindbrain progenitor cells and performed global 5hmC profiles in each group (Figure 5A). Generally, about 5,421 and 4,624 DhMRs were identified in the midbrain and hindbrain progenitors when compared the WT and Pten^−/−^ ESCs. Most of the peaks are located around the intergenic and promoter regions of the whole genome (Figure 5B). We zoomed in to study the hydroxymethylation of differential regulated genes resulted from the loss of Pten. In the midbrain progenitors, impaired Pten expression resulted in the downregulation of 91.8% DhMRs, representing a significant portion of 5hmC loss. While in the hindbrain progenitors, nearly half of the identified DhMRs showed 5hmC gain by Pten loss (Figure 5B). In line with the results that Pten led to the decrease of neural markers in the midbrain and hindbrain lineage, we also observed dynamic 5hmC loss in either the promoter or gene bodies of these neural differentiation genes (Supplementary Figure S5).

To further correlate the DhMRs changes with gene expression, we examined the transcriptomic profiles between WT and Pten^−/−^ ESCs toward midbrain lineage. Among the DEGs, 1,531 upregulated and 1991 downregulated genes were identified with > 1.5-fold change (Figure 5C). KEGG analysis enriched pathways responsible for these DEGs were closely tied to several neurodegenerative diseases, including Huntington disease (HTT), Parkinson disease (PD), and Alzheimer’s disease (AD), suggesting the important role of Pten in regulating neural development (Figure 5D). When we further co-cluster the DEGs with DhMRs in the midbrain progenitors, about 16 genes contain peaks with 5hmC gain, while the loss of 5hmC appears in 123 DEGs (Figure 5E). Since loss of Pten disrupted neuronal differentiation, we focused on the DEGs with 5hmC loss and found that most of these genes were functioned in cell differentiation, signal transduction, and transcriptional regulation (Figure 5F and Supplementary Figure S6).

### 3.5 Pten^−/−^ disrupted midbrain neuron differentiation through mitochondrial dysfunction

Mitochondria are classically known for the generation of energy in the form of ATP, but a renewed appreciation for this organelle has developed a wide array of functions, ranging from metabolism and redox signaling to gene regulation (Murphy et al., 2016). The importance of mitochondria in governing neurogenesis has been a common fact, particularly given the highly energetic nature of neurons. A switch from anaerobic glycolysis in neural progenitor cells to oxidative phosphorylation in mature neurons is the critical process for providing substantial energy for neurogenesis (Khacho and Slack, 2018). Consistently, we have found an increase of genes involved in the mitochondrial metabolism during neuronal differentiation, suggesting the highly active dynamics of mitochondria during neurogenesis (Supplementary Figure S7).

**FIGURE 6:**
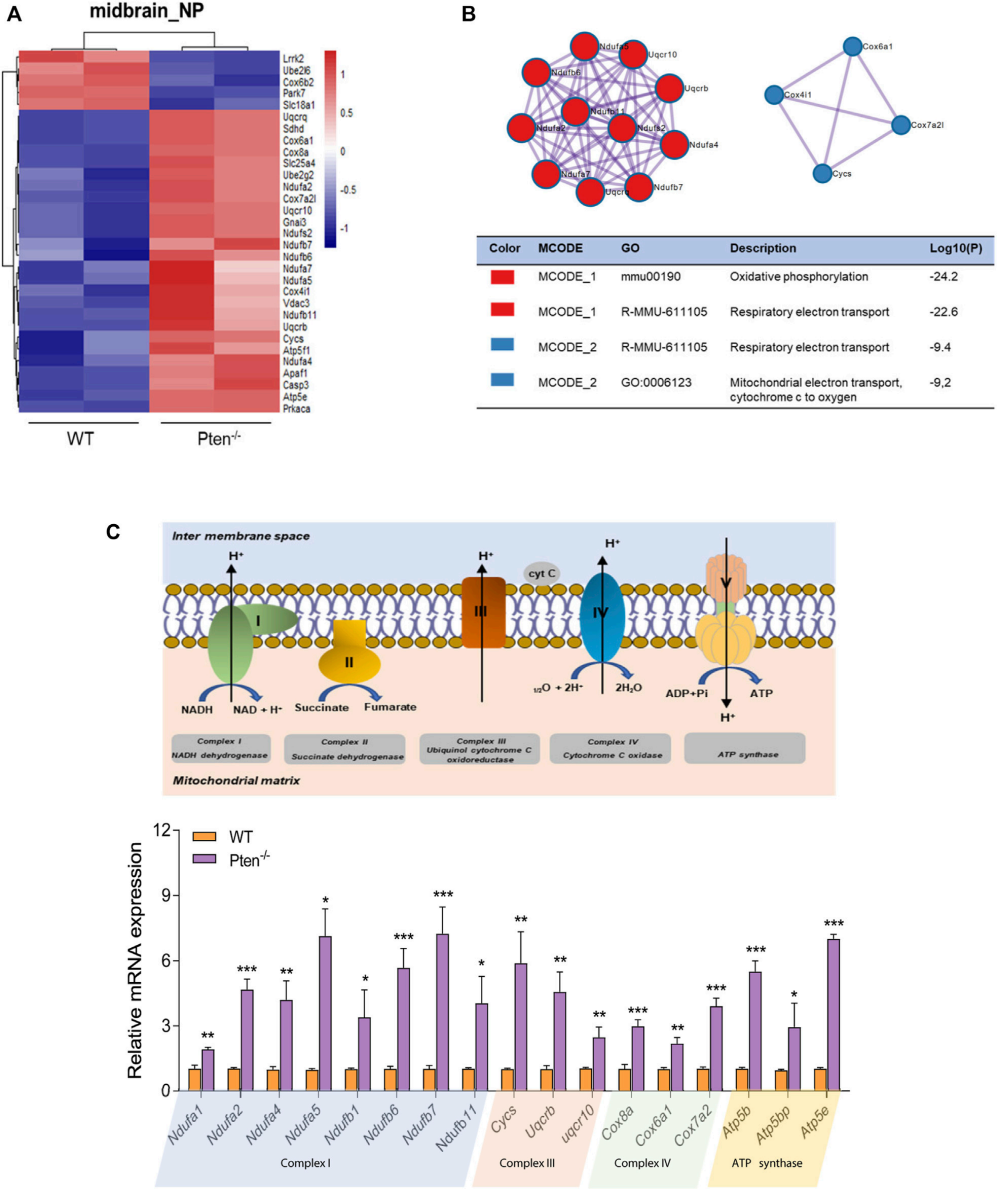
Pten disrupted midbrain differentiation through regulating mitochondrial function. **(A)** Heatmap of mitochondrial associated genes changed by the loss of Pten in midbrain progenitor cells. **(B)** Pathways enriched by Metascape analysis of DEGs involved in mitochondrial function. **(C)** The mRNA expression levels of genes involved in the mitochondrial respiratory chain. *p < 0.05, **p < 0.01, and ***p < 0.001.

It has also been reported that midbrain dopamine neurons are susceptible to gradual mitochondrial damage, therefore justifying one facet of how mitochondrial defects are involved in the etiology of Parkinson’s disease (Liu et al., 2018; Wright, 2022). Consistently, we found that the DEGs involved in midbrain progenitor differentiation pinpointed that mitochondrial associated genes were the most variable genes changed by Pten knockout (Figures 6A,B). Increased expression during differentiation was observed for *Ndufa* and *Ndufb* family members, a set of accessory subunits of mitochondrial respiratory chain complex I (Figure 6C). Consistently, core factors for the mitochondrial respirator chain, such as *Atp5b*, *Cox8a*, and *Cycs* were also upregulated by Pten knockout, suggesting highly active electron transport during midbrain neurogenesis (Figure 6C). We then further examined the 5mC and 5hmC situations of the genes and found that most of the highly active genes showed a 5hmC change, indicating that Pten will impact the mitochondrial metabolism through modulating 5hmC patterning during neurogenesis.

## 4 Discussion

The 5hmC mark is an intermediate state during active DNA demethylation, but it has already been implicated as a functional epigenetic mediator cue critical in governing both normal development and human diseases (Kriaucionis and Heintz, 2009; Cui et al., 2020; Zhang et al., 2020). Several other studies have pioneered to demonstrate how global changes in 5hmC content in specific neural cell types may pattern both embryonic and thereafter postnatal development (Szulwach et al., 2011; Hahn et al., 2013; Kim et al., 2014; Spiers et al., 2017). In this work, we first demonstrated how reassigned DNA hydroxymethylation shall establish a midbrain/hindbrain progenitor transcription program in the immediate, early neural-directed ESC differentiation.

In our study, we characterized the genome-wide landscape of 5hmC in mESC-derived midbrain/hindbrain progenitor cells using a nano-5hmC-seal approach (Han et al., 2016). The conventional bisulfite-based approaches such as TAB-seq or oxBS-seq provide comprehensive and quantitative information due to their ability to detect 5hmC at single-based resolution (Li and Tollefsbol, 2011). However, due to the high sequencing coverage requirements together with frequent risk of genomic DNA degradation, this method was not suitable for samples with limited input available. In particular, enrichment-based profiling methods such as immunoprecipitation requires sufficient inputs up to microgram levels of genomic DNA and have an assay-related tendency to result in base biases, limiting their application in profiling rare stem cell populations, sorted homogenous fractions of specific neuronal cell types, together with skewed preference for cell-free DNA. Therefore, in the current study, we used the nano-5hmC-seal method that includes selective chemical labeling followed by bead capture, providing the required robustness to profile 5hmC in the study of neuronal differentiation using mouse models.

It has been reported that there is a progressive induction of 5hmC levels in both human and murine brain specimens over their entire lifespan and may have implications for neurodegenerative diseases (Spiers et al., 2017). Consistently, we found highly clustered 5hmC in the gene bodies and their promoters, also deposited in real action for brain-specific enhancers, but these marks are depleted naturally from transcription start sites and intergenic regions, both in the midbrain and hindbrain progenitors. Analyses of DhMRs shall reveal their close association with gene bodies that encode neurodevelopmental genes. Gene ontology analyses on these genes that gained 5hmC and active in midbrain/hindbrain neurogenesis confirmed substantial enrichment of genes associated with various neuronal functions, confirming the role of 5hmC in the neurodevelopment. In fact, the same finding was already demonstrated in a prior study in the mouse cerebellum, where 5hmC would increase in both the active gene promoter and its body (Szulwach et al., 2011).

The Tet enzymes are reported to be critical for mammalian development, and loss of these enzymes in ESCs compromises differentiation and embryonic lethality (MacArthur and Dawlaty, 2021). It has been suggested that Tet1 has dual functions, either in maintaining the expression of active genes or in tethering to other cofactors to suppress gene expression. In our study, we observed decreased levels of Tet1 and Tet2 during differentiation in both the midbrain and hindbrain lineages, while the level of Tet3 increased. The Tet1 expression was also reported to be downregulated when human ESCs undergo differentiation towards a neuronal lineage (Kim et al., 2014). Furthermore, Tet3 was found to be increased during neural differentiation. Knockdown of Tet3 led to a genome-wide loss of DNA methylation and hypermethylation of CpGs, which reside in neurogenesis-associated genes (Santiago et al., 2020). As a result, it was believed that the presence of higher 5hmC in a collection of gene bodies corresponds to higher gene expression, those shall recruit TET enzymes locally therefore culminates to transcriptional permissive chromatin along brain development.

PTEN is a classical tumor suppressor renowned in cancer biology, but it has also been named in several publications as an essential factor governing embryonic development (Di Cristofano et al., 1998; Lee et al., 2018). Interestingly, we found that expression of Pten increased in mESC-derived midbrain/hindbrain progenitors. Indeed, earlier research from our lab had already demonstrated that Pten shall maintain the pluripotency of the embryonic stem cell through modulation of GSK3β (Wang et al., 2020). In this study, as an aftermath to the knockout effect of Pten in mESC, we found that neurogenesis would be further impaired during differentiation of both midbrain and hindbrain progenitors. We then further characterized the 5hmC and transcriptomic profiles of mESC-derived midbrain progenitors with Pten deletion. A set of DhMRs/DEGs were associated with neurodegenerative diseases, and pathways involved in the developmental process were enriched, indicating the critical role of Pten partaken in neurogenesis.

Furthermore, an increase in mitochondrial fragmentation in the neurodegenerative disease also highlights the importance of mitochondrial dynamics in neuronal function and survival (Khacho and Slack, 2018). Beckervordersandforth et al. has reported that adult hippocampal neurogenic lineage is critically dependent on mitochondrial electron transport chain and oxidative phosphorylation machinery (Beckervordersandforth et al., 2017). Consistently, we found an increase of genes accounting for mitochondrial metabolism and regulation in both the midbrain and hindbrain progenitors, linking mitochondrial function to efficient lineage progression of NSCs. In addition, Parkinson’s disease is a complex disorder resulting from a combination of both genetic and environment-related factors (Veldman et al., 1998), while mitochondrial impairment plays a central role in dopaminergic neurodegeneration (Filograna and Lee, 2021; Wright, 2022). A recent study has revealed that activation of mitochondrial dysfunction triggered the onset of an immune response and precedes a cascade of organelle failure processes that resulted in cell death (Filograna and Lee, 2021). Our previous study had clearly demonstrated that overexpression of the miR-10 family triggered malfunctioning of mitochondria, thus disrupting the caudalization of floor plate neural progenitors in human iPSCs (Wang et al., 2021).

The Pten insufficiency also reported to result in an increase of mitochondrial complex activity, with activation of PI3K/Akt pathway in the Pten haplo-insufficient mice models (Napoli et al., 2012). When we further mined the genes specifically clustered from the DEGs and DhMRs in midbrain progenitors, a set of genes responsible for mitochondrial functions were enriched along Pten loss, implicating Pten as the modulator on mitochondria regulation. Our subsequent validation had further demonstrated that loss of Pten impaired the expression of genes associated with mitochondria respiratory chain, leading to the abnormal midbrain differentiation.

## 5 Conclusion

In summary, our findings illustrate how early differentiation of both midbrain and hindbrain progenitor cells shall reassign genome-wide 5hmC levels. Such 5hmC alteration is strongly correlated with the activation of a directed neuronal transcriptional program. In addition, Pten was identified as a key regulatory factor responsible for brain neurogenesis, relevant to a dynamic epigenetic regulation of the mitochondrial associated pathways.

## Data Availability

The data presented in the study are deposited in the NCBI repository, accession number PRJNA911849; The data is accessible with this links: https://www.ncbi.nlm.nih.gov/bioproject/PRJNA911849.
