# Supplementary material for: Genome-wide 5-hydroxymethylcytosine (5hmC) reassigned in Pten-depleted mESCs along neural differentiation
Source: Front Cell Dev Biol. 2022 Dec 22;10:956604. doi: 10.3389/fcell.2022.956604 (PMC9814970; doi:10.3389/fcell.2022.956604)
Supplement: Supplementary file 1 [file Table1.DOCX]

**Figure 1**

- Data point for RT-PCR results

|  | **midbrain** | | | |  | **hindbrain** | | | |
| --- | --- | --- | --- | --- | --- | --- | --- | --- | --- |
|  | D0 | D3 | D7 | D14 |  | D0 | D3 | D7 | D14 |
| *Nestin* | 1.201 | 1.124 | 2.874 | 6.545 | *Nestin* | 1.025 | 3.445 | 5.711 | 12.299 |
|  | 1.756 | 1.864 | 3.001 | 6.423 |  | 1.005 | 2.474 | 4.658 | 10.346 |
|  | 0.343 | 1.028 | 3.129 | 6.192 |  | 0.969 | 2.403 | 7.705 | 8.357 |
| *Foxa2* | 0.89 | 8.965 | 66.711 | 190.231 | *Foxa2* | 0.983 | 7.644 | 53.289 | 157.485 |
|  | 1.109 | 9.588 | 63.603 | 186.663 |  | 1.104 | 8.254 | 52.993 | 169.856 |
|  | 1.013 | 9.24 | 58.499 | 186.353 |  | 0.883 | 5.121 | 43.127 | 158.797 |
| *Lmx1a* | 0.96 | 14.206 | 140.942 | 217.808 | *Tubb3* | 0.986 | 2.495 | 9.873 | 15.385 |
|  | 0.982 | 14.361 | 153.061 | 211.784 |  | 0.981 | 3.258 | 5.556 | 24.851 |
|  | 1.061 | 14.564 | 154.557 | 213.559 |  | 1.034 | 4.153 | 8.927 | 24.632 |
| *Otx2* | 0.996 | 3.367 | 5.842 | 13.591 | *Hoxa1* | 1.428 | 8.328 | 11.868 | 17.435 |
|  | 1.016 | 2.184 | 6.608 | 10.484 |  | 1.655 | 7.767 | 15.826 | 19.159 |
|  | 0.989 | 1.847 | 5.715 | 11.942 |  | 0.864 | 5.823 | 12.297 | 10.254 |
| *Nurr1* | 0.993 | 0.945 | 1.842 | 3.591 | *Zic1* | 0.978 | 7.144 | 12.873 | 40.385 |
|  | 1.074 | 1.184 | 1.209 | 3.485 |  | 0.993 | 3.158 | 11.945 | 45.285 |
|  | 0.967 | 1.248 | 1.715 | 4.949 |  | 1.024 | 6.153 | 12.596 | 50.286 |
| *Th* | 0.995 | 0.759 | 1.71 | 2.145 | *Tph1* | 0.893 | 0.552 | 1.064 | 2.112 |
|  | 1.016 | 0.785 | 1.877 | 4.295 |  | 1.108 | 0.834 | 1.259 | 2.351 |
|  | 0.988 | 1.148 | 1.27 | 3.33 |  | 1.004 | 1.191 | 1.217 | 2.745 |
|  |  |  |  |  |  |  |  |  |  |
|  | D0 | D7 | D14 |  |  | D0 | D7 | D14 |  |
| *Tet1* | 1.084 | 1.195 | 0.282 |  |  | 1.008 | 1.139 | 0.327 |  |
|  | 0.897 | 1.213 | 0.456 |  |  | 0.863 | 1.2 | 0.428 |  |
|  | 0.987 | 1.258 | 0.385 |  |  | 1.284 | 0.984 | 0.357 |  |
| *Tet2* | 1.032 | 0.425 | 0.128 |  |  | 1.003 | 0.525 | 0.118 |  |
|  | 0.993 | 0.577 | 0.284 |  |  | 0.852 | 0.648 | 0.204 |  |
|  | 0.934 | 0.589 | 0.278 |  |  | 1.118 | 0.502 | 0.184 |  |
| *Tet3* | 1.104 | 4.295 | 10.628 |  |  | 0.987 | 3.085 | 6.753 |  |
|  | 0.889 | 3.875 | 8.498 |  |  | 0.846 | 2.586 | 7.491 |  |
|  | 0.991 | 4.45 | 11.485 |  |  | 1.194 | 3.586 | 7.784 |  |

- Immunostaining photos

**Nestin-midbrain Nestin_hindbrain**


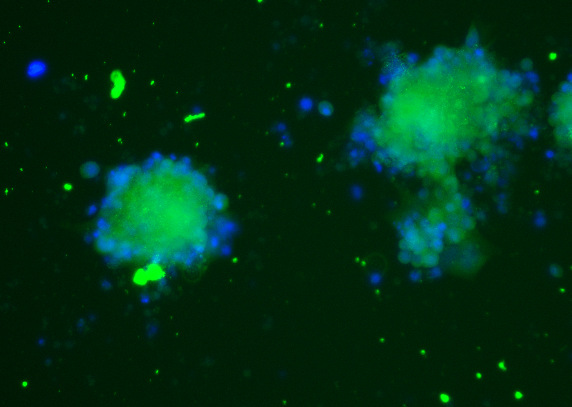

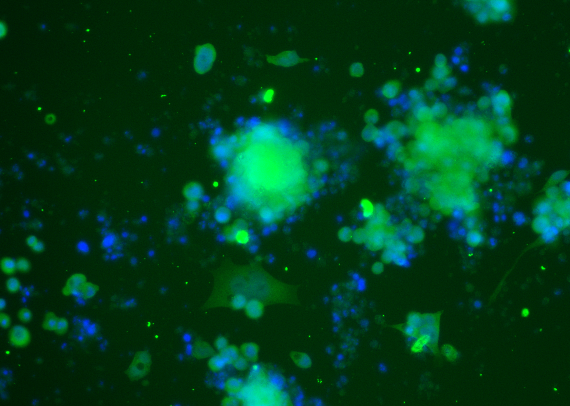


**Foxa2-midbrain Foxa2_hindbrain**


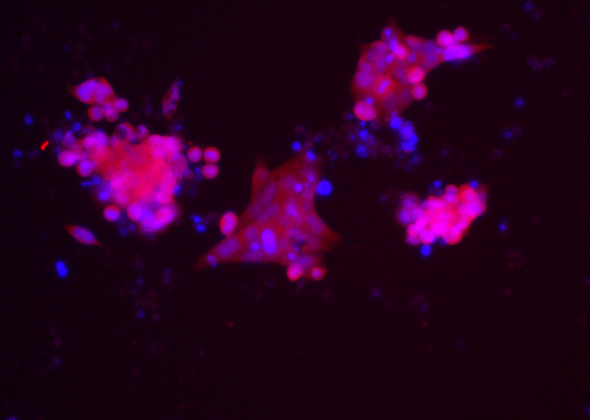

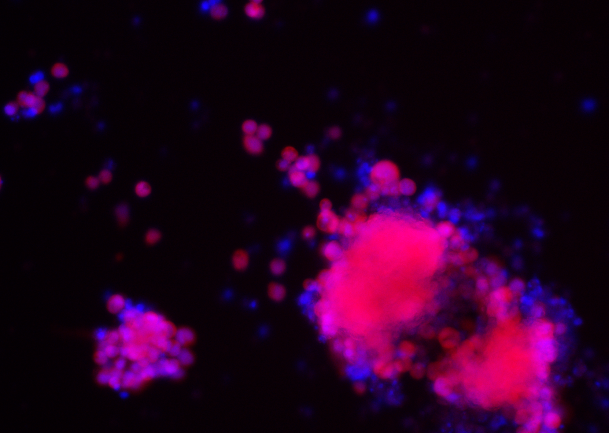


**Lmx1a-midbrain Zic1_hindbrain**


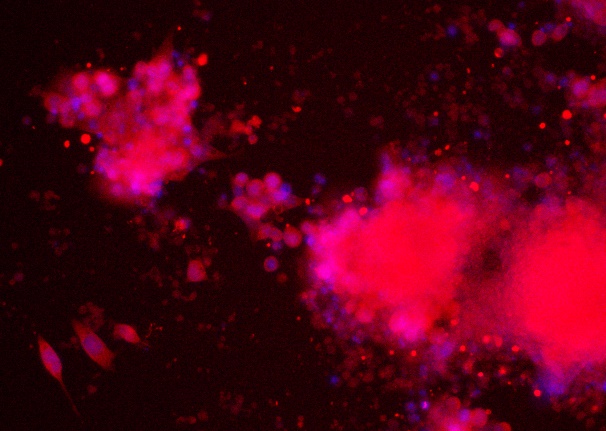

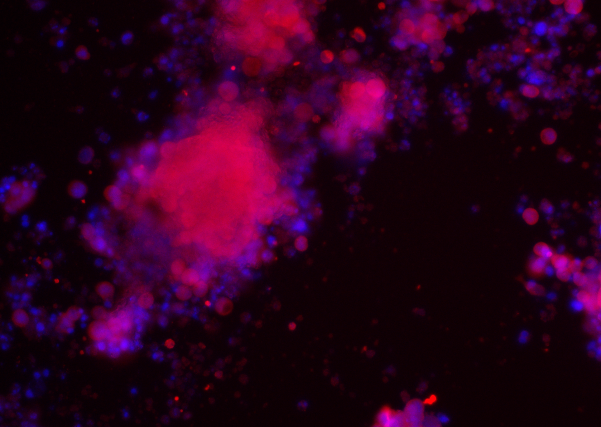


**Otx1/2-midbrain Tuj_hindbrain**


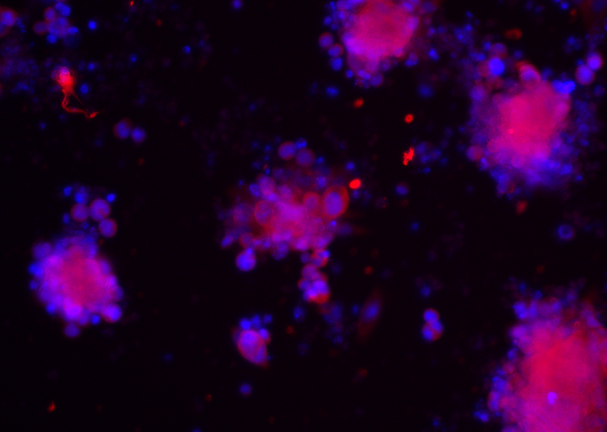

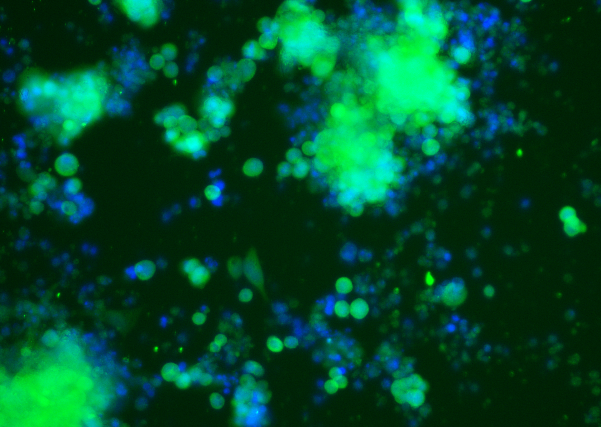


**Figure 4**

- Pten expression RT-PCR

|  | midbrain | | | hindbrain | | |
| --- | --- | --- | --- | --- | --- | --- |
| D0 | 1.022 | 0.938 | 1.03 | 0.976 | 1.034 | 1.044 |
| D3 | 1.624 | 2.553 | 1.729 | 0.842 | 1.424 | 1.256 |
| D7 | 5.566 | 7.538 | 4.559 | 3.135 | 5.341 | 2.463 |
| D14 | 8.249 | 7.596 | 5.951 | 4.242 | 5.248 | 4.837 |

- Neural marker expression

| **midbrain** | **WT** | | | **Pten^-/-^** | | |
| --- | --- | --- | --- | --- | --- | --- |
| *Foxa2* | 0.928 | 1.026 | 1.064 | 0.888 | 1.034 | 1.089 |
|  | 24.542 | 25.378 | 25.919 | 7.387 | 7.734 | 7.414 |
|  | 443.826 | 460.349 | 487.944 | 157.014 | 157.153 | 156.893 |
| *Nestin* | 0.917 | 1.057 | 1.032 | 0.978 | 0.939 | 1.088 |
|  | 0.895 | 0.774 | 0.799 | 2.697 | 2.848 | 2.767 |
|  | 5.138 | 4.665 | 4.393 | 0.587 | 0.535 | 0.467 |
| *Lmx1a* | 1.372 | 0.993 | 0.733 | 1.009 | 1.071 | 0.926 |
|  | 31.152 | 25.256 | 29.279 | 8.981 | 8.367 | 9.096 |
|  | 79.614 | 82.844 | 80.065 | 17.598 | 17.205 | 18.351 |
| *Otx2* | 0.996 | 1.016 | 0.989 | 1.017 | 0.948 | 1.037 |
|  | 2.367 | 2.379 | 2.441 | 0.395 | 0.369 | 0.361 |
|  | 5.842 | 5.609 | 5.715 | 1.631 | 1.466 | 1.558 |
| *Th* | 0.954 | 1.118 | 0.984 | 1.048 | 1.147 | 0.874 |
|  | 0.994 | 1.0745 | 0.986 | 0.997 | 1.084 | 0.846 |
|  | 3.084 | 1.845 | 2.977 | 1.082 | 0.397 | 1.294 |
| **hindbrain** | **WT** | | | **Pten^-/-^** | | |
| *Foxa2* | 0.89 | 1.108 | 1.013 | 1.059 | 0.929 | 1.015 |
|  | 33.489 | 37.842 | 41.495 | 10.813 | 16.294 | 20.184 |
|  | 160.451 | 158.384 | 144.394 | 13.591 | 29.385 | 45.355 |
| *Nestin* | 0.894 | 1.104 | 0.987 | 0.882 | 1.106 | 1.074 |
|  | 3.576 | 6.395 | 3.892 | 1.084 | 1.586 | 1.599 |
|  | 12.845 | 13.742 | 13.997 | 1.486 | 1.134 | 0.948 |
| *Tubb3* | 0.985 | 0.931 | 1.014 | 1.103 | 0.971 | 0.894 |
|  | 3.986 | 5.384 | 4.185 | 1.983 | 2.184 | 0.893 |
|  | 28.485 | 27.953 | 26.105 | 3.566 | 5.395 | 6.993 |
| *Zic1* | 0.936 | 0.981 | 1.034 | 1.194 | 0.958 | 0.884 |
|  | 7.566 | 6.973 | 8.047 | 5.284 | 3.184 | 4.987 |
|  | 60.284 | 75.388 | 77.808 | 2.194 | 0.897 | 1.578 |
| *Tph1* | 1.204 | 0.807 | 0.977 | 0.935 | 1.052 | 1.004 |
|  | 0.998 | 1.004 | 1.023 | 0.853 | 0.934 | 0.573 |
|  | 2.295 | 1.974 | 2.856 | 0.145 | 0.578 | 0.345 |

**Figure 6**

- Mitochondrial respiratory chain gene expression

|  | WT | | | Pten^-/-^ | | |
| --- | --- | --- | --- | --- | --- | --- |
| *Ndufa1* | 0.894 | 1.204 | 0.975 | 1.857 | 1.865 | 2.028 |
| *Ndufa2* | 0.987 | 1.048 | 1.076 | 4.124 | 4.865 | 5.028 |
| *Ndufa4* | 0.875 | 1.139 | 0.957 | 3.596 | 5.194 | 3.805 |
| *Ndufa5* | 1.044 | 0.947 | 0.938 | 7.124 | 8.384 | 5.896 |
| *Ndufb1* | 0.958 | 1.038 | 1.028 | 2.586 | 2.745 | 4.856 |
| *Ndufb6* | 1.034 | 1.135 | 0.895 | 6.395 | 4.682 | 5.94 |
| *Ndufb7* | 0.947 | 0.896 | 1.202 | 8.385 | 5.95 | 7.385 |
| Ndufb*11* | 0.973 | 1.048 | 1.054 | 5.466 | 3.295 | 3.367 |
| *Cycs* | 0.976 | 1.045 | 1.004 | 4.25 | 6.496 | 6.93 |
| *Uqcrb* | 0.891 | 1.194 | 0.945 | 3.586 | 5.384 | 4.722 |
| *uqcr10* | 0.993 | 1.083 | 1.058 | 2.874 | 1.957 | 2.579 |
| *Cox8a* | 0.884 | 1.254 | 0.903 | 3.297 | 2.685 | 2.945 |
| *Cox6a1* | 1.084 | 1.003 | 0.946 | 1.956 | 2.495 | 2.086 |
| *Cox7a2* | 1.104 | 0.945 | 1.0375 | 4.295 | 3.856 | 3.579 |
| *Atp5b* | 0.974 | 1.105 | 0.992 | 5.94 | 4.96 | 5.594 |
| *Atp5bp* | 0.994 | 0.893 | 0.962 | 2.295 | 2.294 | 4.224 |
| *Atp5e* | 1.085 | 0.964 | 1.033 | 7.234 | 6.948 | 6.84 |

**Coding Script**

- **R script – 5hmC analysis**

$ fastqc sample1_R1.fastq.gz

trim_galore -q 20 --phred33 --stringency 3 --length 20 -e 0.1 --paired (file1) (file2) -o (~dir)

$ bowtie2 -x (index) -1 (file1) -2 (file2) -S (name.sam)

$ samtools view -bS sample.sam > sample.bam; samtools sort sample.bam -o sample.sorted.bam; samtools index sample.sorted.bam; ambamba markdup -r (sample.sorted.bam)

$ bamCoverage -e 170 -bs 10 -b sample.sorted.bam -o sample.bw

$ macs2 callpeak -f BAM -c control.bam -t sample.bam $i -n $i -g hs --outdir ../macs2/ --bdg -q 0.05

$ computeMatrix reference-point -p 15 --referencePoint TSS -b 5000 -a 5000 -R mm10_bedfile -S sample.bw --skipZeros -o sample.gz --outFileSortedRegions sample_genes.bed

$ plotHeatmap -m sample_genes.gz -out sample.heatmap.png --colorMap RdBu --whatToShow 'heatmap and colorbar' --zMin -10 --zMax 10

$ manorm --p1 sample1_peaks.bed --p2 sample2_peaks.bed --r1 sample1_reads.bed --r2 sample2_reads.bed --n1 name1 --n2 name2 -o output_dir

- **R script – RNAseq analysis**

$ fastqc sample1_R1.fastq.gz

$ trim_galore -q 20 --phred33 --stringency 3 --length 20 -e 0.1 --paired file1 file2 -o filename

$ STAR --runThreadN 20 --runMode genomeGenerate --genomeDir (destination file) --genomeFastaFiles (Mus_musculus.GRCm39.dna.primary_assembly.fa.gz) --sjdbGTFfile (Mus_musculus.GRCm39.104.chr.gtf) --sjdbOverhang 149

$ RUN STAR --genomeDir \

--readFilesIn ~/File destination \

--readFilesCommand zcat \

--outSAMtype BAM SortedByCoordinate \

--quantMode GeneCounts \

--outFileNamePrefix alignments_STAR

--runThreadN 4

$ rsem-prepare-reference –gtf (gtf file destination) \

--trusted-sources BestRefSeq,Curated\ Genomic \

--STAR \

mouse_assembly.fna \

~ (index destination)

rsem-extract-reference-transcripts ~ (index destination) ~ (mouse.gtf) ~ (mouse_primary_assembly.fna)

$ rsem-generate-data-matrix *.gene.results > out_matrix
