# Supplementary material for: Genome-wide 5-hydroxymethylcytosine (5hmC) reassigned in Pten-depleted mESCs along neural differentiation
Source: Front Cell Dev Biol. 2022 Dec 22;10:956604. doi: 10.3389/fcell.2022.956604 (PMC9814970; doi:10.3389/fcell.2022.956604)
Supplement: Supplementary file 2 [file DataSheet1.PDF]

Supplementary Material

Genome-wide 5-hydroxymethylcytosine (5hmC) reassigned in Pten-depleted mESCs along neural differentiation

Zhangting Wang[1], Kai-Kei Miu[1#], See-Wing Chan[1], Fang-Hong Ou[1], Patrick Wai-Nok Law[1], Wai-Yee Chan[1,2#]

[1] School of Biomedical Sciences, Faculty of Medicine, The Chinese University of Hong Kong, Hong Kong SAR, Hong Kong SAR, China.

[2] Key Laboratory for Regenerative Medicine (Jinan University-The Chinese University of Hong Kong), Ministry of Education, School of Biomedical Sciences, Faculty of Medicine, The Chinese University of Hong Kong, Hong Kong SAR, China.

Corresponding Author:

Kai-Kei Miu, Ph.D

Rm125, Lo Kwee-Seong Integrated Biomedical Sciences Building, Area 39,

The Chinese University of Hong Kong, Hong Kong SAR, Hong Kong, China.

Phone: (852) 3943 0570

E-mail: kelvinmiu@cuhk.edu.hk;

Wai-Yee Chan, Ph.D

Rm 123A, Lo Kwee-Seong Integrated Biomedical Sciences Building, Area 39,

The Chinese University of Hong Kong, Hong Kong SAR, Hong Kong, China.

Phone: (852) 3943 1383; Fax: (852) 2603 7902

E-mail: chanwy@cuhk.edu.hk;

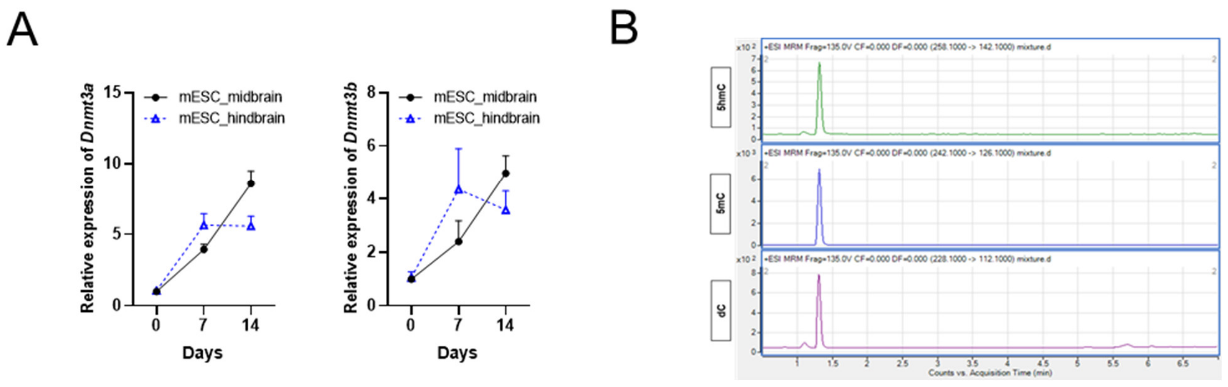

Supplementary Fig. S1. (A) The mRNA expression of Dnmt3a and Dnmt3b in mESC-derived midbrain and hindbrain progenitor cells. (B) Representative images of 5hmC, 5mC and dC standards detected by LC-MS/MS.

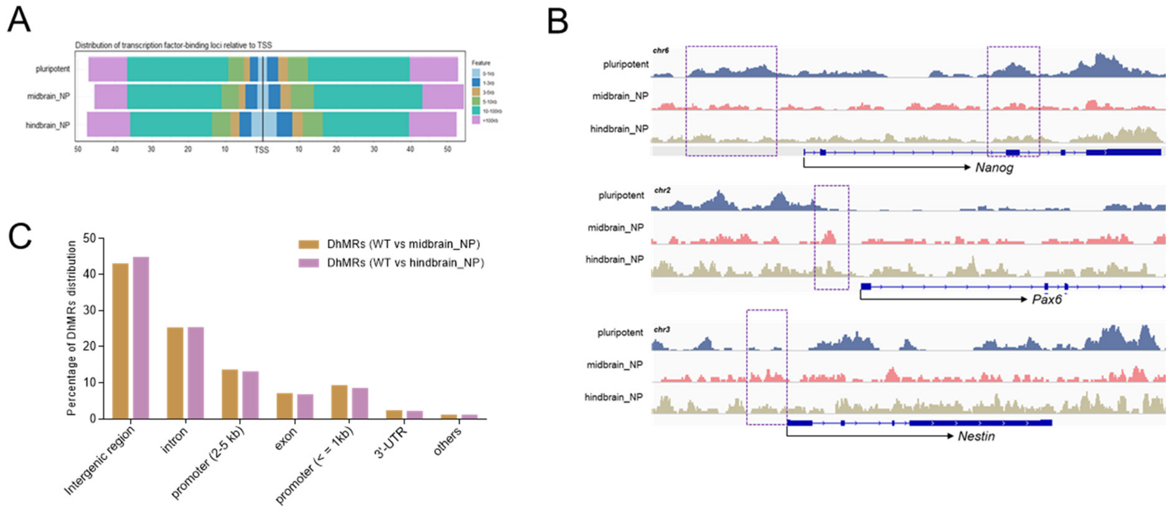

Supplementary Fig. S2. (A) Distribution of transcription factor-binding loci relative to TSS. (B) The Genome Browser visualization of 5hmC patterns of pluripotency marker (*Nanog*) and neural differentiation markers (*Pax6* and *Nestin*) in pluripotent mESC and midbrain & hindbrain progenitors. (C) Genomic distribution of the differentially hydroxymethylated regions (DhMRs) identified in midbrain_NP vs ESCs and hindbrain_NP vs ESCs.

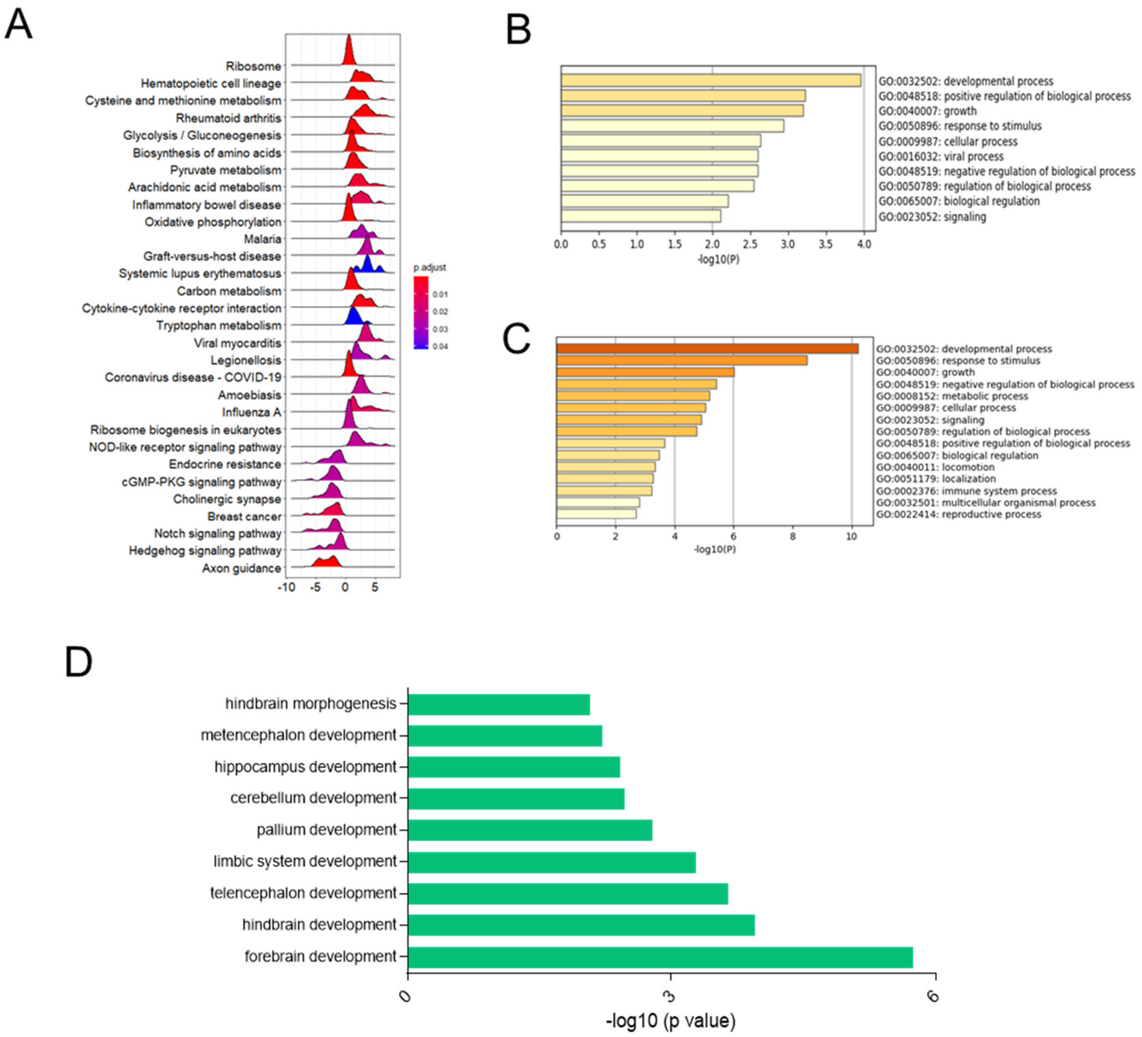

Supplementary Fig. S3. (A) Top enriched Go terms involved in the mESC at day 6 after neural differentiation. (B) The top-level Gene Ontology biological processes enriched from genes clustered from DhMRs in midbrain progenitors and DEGs. (C) The top-level Gene Ontology biological processes enriched from genes clustered from DhMRs in hindbrain progenitors and DEGs. (D) Bar graph of enriched terms subject to head development.

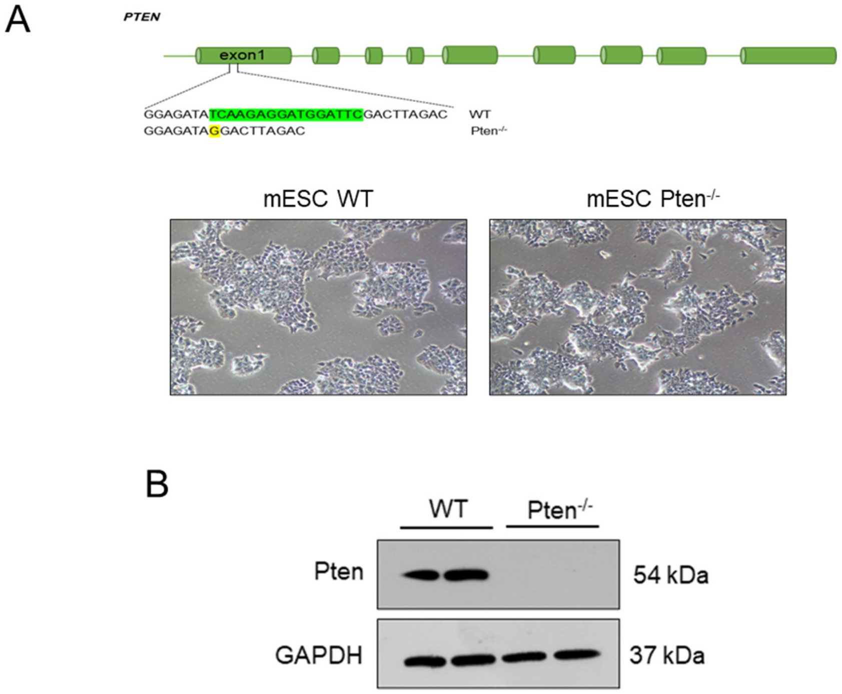

Supplementary Fig. S4. (A) The *Pten* deletion in mouse ESCs by CRISPR-Cas9. Representative phase-contrast images of wild-type (WT) and Pten$^{-/-}$ ESCs. (B) Immunostaining of Pten in the wild type and Pten$^{-/-}$ mouse ESCs. GAPDH were used as the loading control.

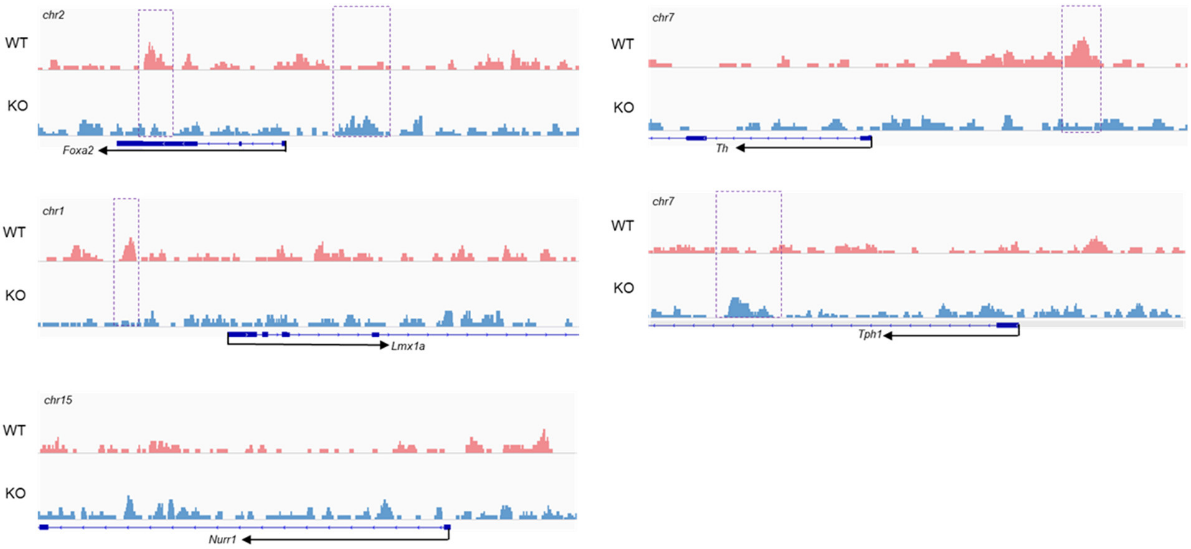

Supplementary Fig. S5. The Genome Browser visualization of 5hmC patterns in neural differentiation markers including Foxa2, Lmx1a, Nurr1, Th and Tph1 in the midbrain progenitors derived from wild type and Pten$^{-/-}$ mESCs.

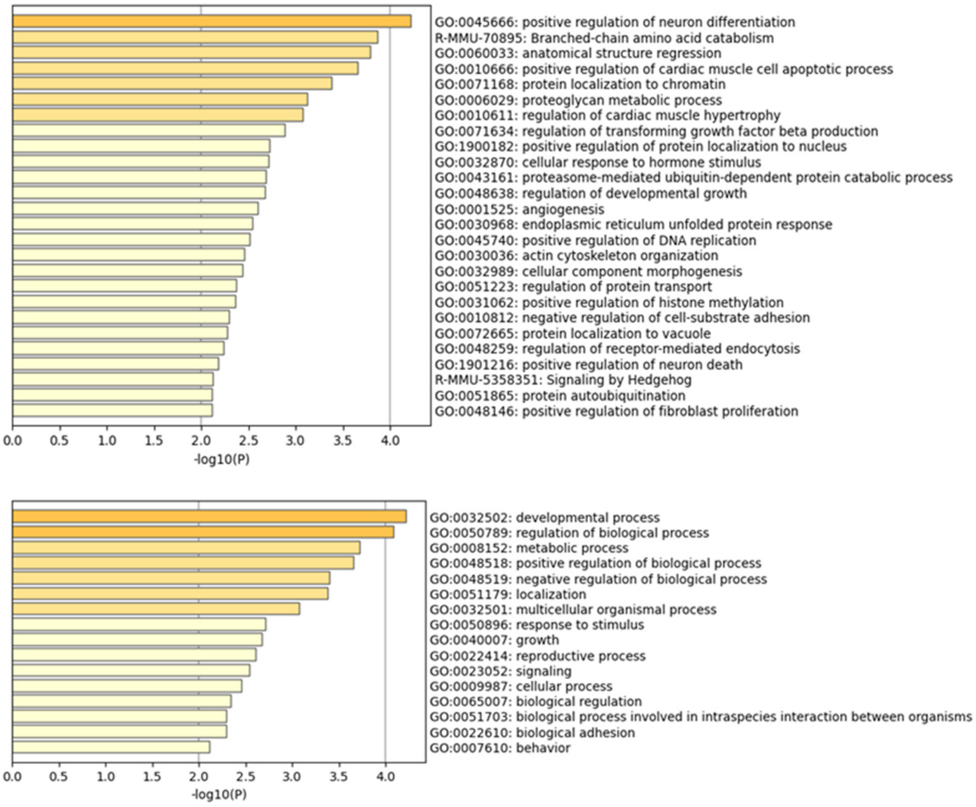

Supplementary Fig. S6 Top enriched Go terms of genes clustered from DEGs and DhMRs with 5hmC loss in WT and Pten$^{-/-}$ ESC-derived midbrain progenitor cells.

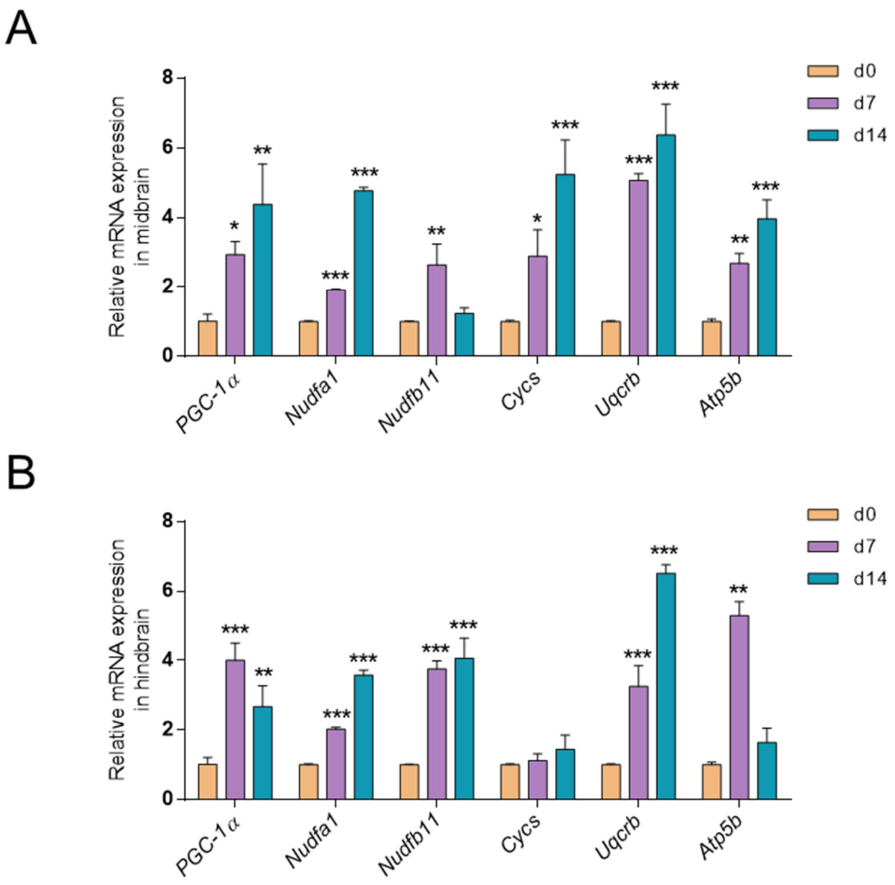

Supplementary Fig. S7 The mRNA expression of genes involved in mitochondrial regulation and metabolism in midbrain and hindbrain specification. All data are expressed as mean ± SD (n=3). *$p <$ 0.05, **$p < 0.01$, and ***$p < 0.001$.

Supplementary Table S1. Primer sequences of target genes

Primers		Sequences
Gapdh	Up (5’-3’)	TGGTGAAGCAGGCATCTGAG
	Down (5’-3’)	TGAAGTCGCAGGAGACAACC
Nestin	Up (5’-3’)	CTGCAGGCCACTGAAAAGTT
	Down (5’-3’)	TCTGACTCTGTAGACCCTGCTTC
Foxa2	Up (5’-3’)	AAGTAGCCACCACACTTCAGG
	Down (5’-3’)	TGTGGCCCATCTATTTAGGG
Lmx1a	Up (5’-3’)	ACCGTTCCTCCGCTCTACA
	Down (5’-3’)	CTTCAGGCCGTCCAACAT
Otx2	Up (5’-3’)	GGTATGGACTTGCTGCATCC
	Down (5’-3’)	CTCTCCCTTCGCTGTTTCC
Nurr1	Up (5’-3’)	TCAGAGCCCACGTCGATT
	Down (5’-3’)	TAGTCAGGGTTTGCCTGGAA
Th	Up (5’-3’)	GGACCACCAGCTTGCACTAT
	Down (5’-3’)	GTATCCTGCTCTGAGACGGC
Tubb3	Up (5’-3’)	GCGCATCAGCGTATACTACAA
	Down (5’-3’)	TTCCAAGTCCACCAGAATGG
Hoxa1	Up (5’-3’)	CCACTCGAGTTGTGGTCCAA
	Down (5’-3’)	CCCACCACTTACGTCTGCTT
Zic1	Up (5’-3’)	GCTATCAGTCTCGCGCTCA
	Down (5’-3’)	TCTTTTGCGGTTTATCTTCCTG

Tph1	Up (5’-3’)	AGACTAAAGCAGTCTTGCCTGG
	Down (5’-3’)	TCAGTTAACAGCCCCCATGTC
Tet1	Up (5’-3’)	TGGTGGCGTATAGCACAGTT
	Down (5’-3’)	CCCCAAGGTCTAGGATGGGA
Tet2	Up (5’-3’)	CCATCATGTTGTGGGACGGA
	Down (5’-3’)	TCTGAGAACAGCGACGGTTG
Tet3	Up (5’-3’)	GGGCAGGCAGCGTAGC
	Down (5’-3’)	ATGAGGTGAGCCAATGGGTG
Dnmt3a	Up (5’-3’)	CTGCTGTGGAATACCCTGTTAG
	Down (5’-3’)	CTTTCTACCTGCTGCCATACTC
Dnmt3b	Up (5’-3’)	GGTCTCCAGCCTTCTGAATTAC
	Down (5’-3’)	CAGAGCCATTCCCATCATCTAC
Pten	Up (5’-3’)	CAGTGAATGCCATCACCATTTC
	Down (5’-3’)	CTGGGCTTAAGGTCTGATTCTC
Ndufa1	Up (5’-3’)	AGAGAGGTAAAGCCGGGTCA
	Down (5’-3’)	ACATCTCCGCACCGTTACTC
Ndufa2	Up (5’-3’)	TGCTAACAAAGATGGCGGCT
	Down (5’-3’)	CCGTTGCACGATGAAATCCC
Ndufa4	Up (5’-3’)	CGGCTTAGCGTGTGTCCTAA
	Down (5’-3’)	GCCAAGCGCATCACATACAG
Ndufa5	Up (5’-3’)	GGATATGGTCAAGGCGGAGC
	Down (5’-3’)	GCCACTTCCACTGGTTAGCA

Ndufb1	Up (5'-3')	CCAGGCTGAAGCAGTCAAGA
	Down (5'-3')	GACAAATCCCGCAGGGACAA
Ndufb6	Up (5'-3')	GTCGGCAACATGTCAGGGTA
	Down (5'-3')	GCCATCGTCTCCTTAGCTCC
Ndufb7	Up (5'-3')	GGTGACCCCGGCTACTAAAG
	Down (5'-3')	CAGTAGTCCCAGTCGTGCTG
Ndufb11	Up (5'-3')	GCAGCTGCGCACATTGTATT
	Down (5'-3')	GGTTCTCGTTGCCGCTTTTT
Cycs	Up (5'-3')	GAACAAGTGTGGTTGCACCG
	Down (5'-3')	TAATTCGTTCCGGGCTGGTC
Uqcrb	Up (5'-3')	TTAAGAGAGCCCTGGACCTGA
	Down (5'-3')	AGGCACACCCACAGATCTTA
Uqcr10	Up (5'-3')	ATCAACGAGGGGAAACTGTGG
	Down (5'-3')	TGAACGGCAACTTGAAACTCAT
Cox8a	Up (5'-3')	ACAAGAAGCGGGAGTGAAGG
	Down (5'-3')	CCCACCAAGCAGAGCCAATA
Cox6a1	Up (5'-3')	AGCACTGGTTTTGGACCCTT
	Down (5'-3')	GTCGCCATCTGAGGAAAGGA
Cox7a2	Up (5'-3')	AGTTCATCTGAAAGGCGGGG
	Down (5'-3')	TGAACCACGTGAAGACTGGG
Atp5b	Up (5'-3')	GTTGGTCCTGAGACCTTGGG
	Down (5'-3')	TCCGATTTTCCCACCCTTGG
Atp5bp	Up (5'-3')	GACAGGCTGGACTCAGCTAC

	Down (5'-3')	CCCGAAGTCTTCTCAGCGTT
Atp5e	Up (5'-3')	GACAGGCTGGACTCAGCTAC
	Down (5'-3')	CCCGAAGTCTTCTCAGCGTT
